# Nanocomposites of Conducting Polymers and 2D Materials for Flexible Supercapacitors

**DOI:** 10.3390/polym16060756

**Published:** 2024-03-09

**Authors:** Haipeng Zhu, Ruiqi Xu, Tao Wan, Wenxiong Yuan, Kewei Shu, Natkritta Boonprakob, Chen Zhao

**Affiliations:** 1 School of Materials and Energy, Guangdong University of Technology, Guangzhou 510006, China; zhp1162465894@163.com (H.Z.); 17620946254@163.com (R.X.); wt549294345@163.com (T.W.); 13202820987@163.com (W.Y.); 2 Xi’an Key Laboratory of Advanced Performance Materials and Polymers, Shaanxi Key Laboratory of Chemical Additives for Industry, Shaanxi University of Science and Technology, Xi’an 710021, China; 3 Program of Chemistry, Faculty of Science and Technology, Uttaradit Rajabhat University, Uttaradit 53000, Thailand; natkritta.boo@uru.ac.th

**Keywords:** conducting polymer, 2D materials, flexible, supercapacitor, integrated

## Abstract

Flexible supercapacitors (FSCs) with high electrochemical and mechanical performance are inevitably necessary for the fabrication of integrated wearable systems. Conducting polymers with intrinsic conductivity and flexibility are ideal active materials for FSCs. However, they suffer from poor cycling stability due to huge volume variations during operation cycles. Two-dimensional (2D) materials play a critical role in FSCs, but restacking and aggregation limit their practical application. Nanocomposites of conducting polymers and 2D materials can mitigate the above-mentioned drawbacks. This review presents the recent progress of those nanocomposites for FSCs. It aims to provide insights into the assembling strategies of the macroscopic structures of those nanocomposites, such as 1D fibers, 2D films, and 3D aerogels/hydrogels, as well as the fabrication methods to convert these macroscopic structures to FSCs with different device configurations. The practical applications of FSCs based on those nanocomposites in integrated self-powered sensing systems and future perspectives are also discussed.

## 1. Introduction

The rise of flexible and wearable electronics has triggered increasing demands for flexible power sources that can provide sufficient energy to ensure continuous and long-term operation under mechanical deformations [1,2,3]. Flexible supercapacitors (FSCs) have been considered to be a promising power source for flexible and wearable electronics in recent years [4]. Similar to conventional supercapacitors, FSCs can deliver energy density with orders of magnitude higher than plate capacitors and a significantly higher power density than batteries [5]. Depending on the charge storage mechanisms, FSCs are mainly categorized into electrical double-layer capacitors (EDLCs) and pseudocapacitors. Carbon materials with a high surface area such as activated carbon, carbon nanotubes, and graphene are widely employed in EDLCs as the charge in EDLCs is stored by the adsorption of electrolyte ions on the electrode surface [6]. Redox-active materials such as transition metal oxides and conducting polymers are predominantly used in pseudocapacitors as the capacitance originates from the fast and reversible redox reactions [7,8].

Over the past decades, pseudocapacitors have attracted significant attention due to their higher specific capacitances than EDLCs. Compared with metal oxides, conducting polymers have been regarded as the most promising pseudocapacitive materials for FSCs due to their high specific capacitance, good conductivity, ease of synthesis, and flexibility [9,10]. Conducting polymers store energy using the reverse doping and de-doping processes, leading to large specific capacitances in common conducting polymers such as 750 F g^−1^ for polyaniline (PANi), 620 F g^−1^ for polypyrrole (PPy), 485 F g^−1^ for polythiophene (PTh), and 210 F g^−1^ for poly(3,4-ethylenedioxythiophene) (PEDOT) [11,12]. Unfortunately, the poor cycling stability of conducting polymers caused by the volume changes occurring in the doping–de-doping process restricts their practical application [13].

Recently, two-dimensional (2D) materials with atomic or near-atomic thickness have gained significant interest for energy storage due to their unique physicochemical and electrochemical properties [14,15,16,17,18,19]. Graphene [20], transition metal dichalcogenides (TMDs) such as MoS_2_ [21], transition metal carbides, nitrides and carbonitrides (MXenes) [22], and black phosphorus (BP) [23] have been intensively investigated for their application in FSCs. However, these 2D nanosheets tend to restack due to the π–π interaction and van der Waals forces, resulting in a reduced accessible surface area and deteriorated electrochemical performance. Nanocomposites based on conducting polymers and 2D materials can inherit the advantages of each individual part with a synergistic effect to achieve superior performance. The high mechanical strength and flexibility of 2D nanosheets help to improve the structural stability of the backbones of conducting polymers. Meanwhile, conducting polymers can act as the conducting mediate to bridge the 2D nanosheets and prevent the restacking of those nanosheets [24]. 

**Figure 1:**
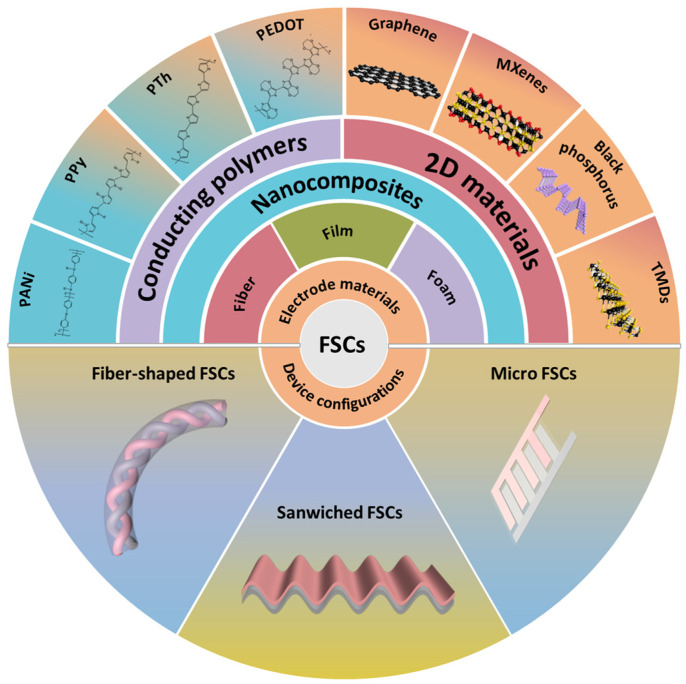
Schematic of electrodes based on nanocomposites of conducting polymers and 2D materials as well as device configurations for flexible supercapacitors.

In this review, the recent progress in the fabrication of FSCs based on nanocomposites of conducting polymers and 2D materials is comprehensively discussed. Initially, the general synthesis routes for conducting polymers and 2D materials are introduced. Then, recent developments in the macroscopic assemblies of those nanocomposites, including 1D fibers, 2D films, and 3D aerogels/hydrogels, are discussed. In addition, flexible supercapacitors based on macroscopic assemblies with various device configurations are summarized in detail (Figure 1). Some integrated self-powered sensing systems with those FSCs are also presented. Finally, future perspectives on nanocomposite developments for FSCs are discussed. 

This review aims to inspire future research endeavors to take the full advantage of the nanocomposites of conducting polymers and 2D materials to fabricate advanced FSCs. The scope of this review is to summarize the assembling strategies of the macroscopic structures of those nanocomposites and the fabrication methodologies for FSCs. This review is expected to bring new insights into the material preparation and configuration design of novel FSCs.

## 2. Nanocomposites of Conducting Polymers and 2D Materials

### 2.1. General Synthesis Routes

Conducting polymers are mainly synthesized either by oxidative chemical polymerization or electrochemical polymerization to achieve various forms such as powders [25], films [26,27,28,29,30], and hydrogels [31,32,33,34,35], with a large variety of morphologies, including (but not limited to) nanofibers [36,37,38,39,40], nanotubes [41,42,43,44,45,46], and nanosticks [47]. In oxidative chemical polymerization, the monomers are oxidized by the oxidants (such as ferric chloride) to form radical cations, followed by the reaction of two radical cations to produce a dimer. Then, re-oxidation and coupling are repeated to form conducting polymers [48]. The main advantage of oxidative chemical polymerization is that it can synthesize conducting polymers in large quantities [10]. In electrochemical polymerization, the monomers are electrochemically oxidized [49]. Compared with chemical polymerization, electrochemical polymerization possesses a number of advantages: it is easy to introduce different dopants, produce layered structures, and form copolymers. 

**Figure 2:**
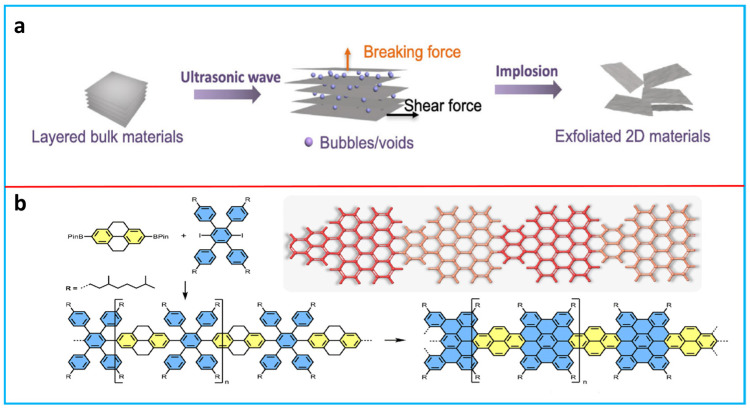
(**a**) Top-down exfoliation of 2D materials using ultrasonication. Adapted with permission from [50]. Copyright 2021, Springer Nature. (**b**) Bottom-up wet chemical synthesis of graphene nanoribbons. Reproduced from [62] with permission from the Royal Society of Chemistry.

A number of preparation methods have been developed for 2D materials, which can be classified into two main categories: top-down exfoliation and bottom-up growing [50]. Successful top-down exfoliation happens when the mechanical forces or ion/molecule intercalations are sufficient enough to overcome the interlayer interactions without damaging the in-plane structures [51]. Mechanical exfoliation breaks the van der Waals attraction between the nanosheets by shear forces, which can be induced by different techniques such as ultrasonication (Figure 2a) [52,53,54], ball milling [55], high-shear mixing [56], and grinding [57]. Graphene, MoS_2_, and BP have been successfully exfoliated through this approach. Ion/molecule intercalation-assisted exfoliation can be regarded as a supplement to the mechanical exfoliation approach. It involves the insertion of ions or molecules into the interlayer spacing of layered bulk crystals, thereby enlarging the interlayer distance and diminishing the van der Waals interactions among the layers. Further ultrasonication using suitable solvents enables the facile exfoliation of single- or few-layer nanosheets from these bulk crystals intercalated with ions/molecules [58,59]. Selective etching plays a critical role in preparing MXene materials. MXene is exfoliated by the selective etching of the A-phase from the MAX-phase using various etchants [60]. The bottom-up approach has been proposed to directly synthesize 2D materials through various precursor-driven chemical reactions, including chemical vapor deposition (CVD) and wet chemical synthesis. CVD can produce highly crystalline 2D materials on substrates under high-vacuum and high-temperature conditions. Large-area graphene single crystals can be obtained on Cu foils by CVD [61]. Wet chemical synthesis involves the formation of 2D materials in a solution. For example, Feng et al. reported the synthesis of curved graphene nanoribbons from tetrahydropyrene-based polyphenylenes in dichloromethane (Figure 2b). The obtained graphene nanoribbons showed a high charge mobility of 3.6 cm^2^ V^−1^ s^−1^ [62]. 

### 2.2. Macroscopic Assemblies of Nanocomposites

When subjected to mechanical deformation, FSCs with powdery active materials may encounter a decrease in electrochemical performance due to the poor adhesion between the current collector and active materials [63]. Thus, flexible electrodes with macroscopic structures (1D fibers, 2D films, and 3D aerogels/hydrogels) are highly favorable to maintain the integrality of the electrodes [64,65]. The macroscopic structures can be assembled with nanocomposites themselves or on diverse substrates. However, most of the substrates are not electroactive [24]; thus, only macroscopic assemblies with nanocomposites themselves as building blocks are discussed here.

#### 2.2.1. Fibers

Fiber electrodes are essential for fiber-shaped supercapacitors with high flexibility. They have attracted significant attention for wearable electronics due to their ability to be integrated with commercial fabrics or garments. Two approaches have been proposed to produce nanocomposite fibers of conducting polymers and 2D materials. One is the direct spinning of the mixture of all components and the other is post-deposition, which involves host fiber formation and subsequent material deposition. 

**Figure 3:**
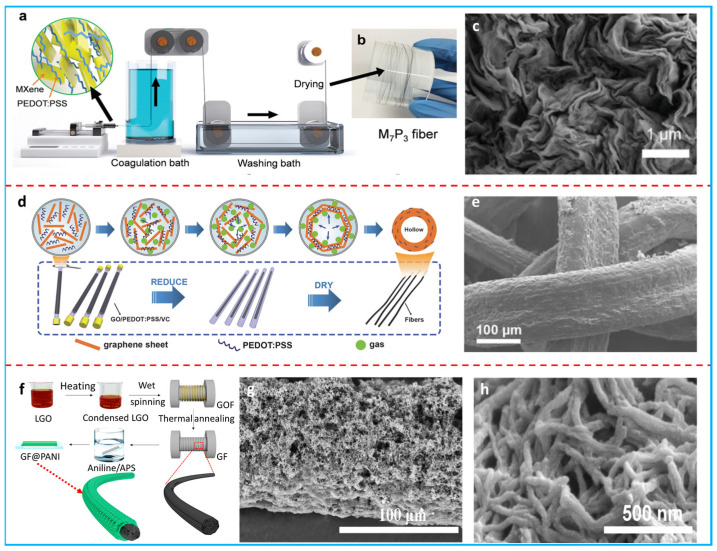
(**a**) Schematic illustration of the wet spinning set-up to produce a PEDOT:PSS/MXene composite fiber. (**b**) Digital photo of a 5 m long PEDOT:PSS/MXene composite fiber. (**c**) SEM image of the cross-section of a PEDOT:PSS/MXene composite fiber. Adapted with permission from [66]. Copyright 2019, Wiley-VCH. (**d**) Schematic illustration of the preparation of a hollow PEDOT:PSS/rGO composite fiber. (**e**) SEM image of a knotted hollow PEDOT:PSS/rGO composite fiber. Adapted with permission from [70]. Copyright 2016, Wiley-VCH. (**f**) Schematic of the fabrication process of PANi nanorods/rGO composite fiber. (**g**,**h**) SEM images of PANi nanorods/rGO composite fiber. Adapted with permission from [73]. Copyright 2019, American Chemical Society.

Spinning is a feasible and scalable method to produce fibers and can be classified into wet spinning and microfluidic hydrothermal spinning. Wet spinning is conducted by injecting spinning dopes into a coagulation bath, causing the fiber to coagulate and solidify. The solvent in the coagulation bath should be a good nonsolvent for both components and the composition of the spinning dopes should be well-tuned to ensure long-fiber production. PEDOT:PSS, MXene, and GO are inherently processable by wet spinning [66,67]. Razal et al. used this spinnability to fabricate PEDOT:PSS/MXene fibers in a H_2_SO_4_ bath (Figure 3a) [66]. Here, PEDOT:PSS acted as the conductive binder to glue the MXene nanosheets together. The MXene loading in the spinning dope was as high as 70 wt%. Continuous fibers over 5 m were obtained with high reproducibility (Figure 3b). MXene sheets were oriented along the fiber direction (Figure 3c). The composite fibers with 70 wt% MXene reached a maximum conductivity of ~1489 S cm^−1^ and exhibited a high strength of ~58.1 MPa as well as a high volumetric capacitance of 614.5 F cm^−3^. They also showed good flexibility and could form a knot. Wang et al. prepared PEDOT:PSS/reduced graphene oxide (rGO) composite fibers using a wet spinning process [67]. A mixed solution of PEDOT:PSS and GO was injected into a coagulation bath of CaCl_2_ to obtain the PEDOT:PSS/GO fibers and then they were immersed in concentrated sulfuric acid to enhance the conductivity of PEDOT:PSS, followed by a vitamin C treatment to reduce the GO. The PEDOT:PSS/rGO composite fibers showed a high conductivity up to ~590 S cm^−1^ and a high strength up to ~18.4 MPa. A highly wrinkled structure was found on the surface, which could increase the specific area to improve electrolyte wetting. The composite fibers showed an areal specific capacitance of 131 mF cm^−2^. Microfluidic hydrothermal spinning is a facile method to prepare fibers through a long and slender tube confined by a hydrothermal process. GO and PEDOT:PSS can be well-adopted in microfluidic spinning to produce composite fibers due to their good gelation ability during the hydrothermal process [68,69,70]. Peng’s group reported the preparation of hollow PEDOT:PSS/rGO composite fibers in sealed glass pipes (Figure 3d) [70]. A mixed solution of GO, PEDOT:PSS, and vitamin C was injected into a glass pipe and then the two ends of the pipe were sealed. The gas released from the reduction in GO pushed the formed rGO sheets from the center to form a hollow structure. The hollow composite fiber was highly flexible and had a rough and wrinkled surface, which could increase the specific area for enhanced capacitance (Figure 3e). The fiber showed a high tensile strength up to 631 MPa, a high conductivity up to 4700 S m^−1^, and a high areal specific capacitance of 304.5 mF cm^−2^.

In the post-deposition strategy, host fibers are generally prepared by spinning, followed by the deposition of other active materials [71,72,73,74,75,76]. rGO fibers are widely explored as the host fibers to conduct polymer deposition due to their high flexibility and high conductivity. Zhang et al. reported an in situ chemical polymerization method to deposit polyaniline nanorods on wet-spun rGO fibers (Figure 3f) [73]. Interconnected PANi nanorods formed the sheath of the composite fiber (Figure 3g,h). The composite fiber showed a high conductivity of 1.4 × 10^4^ S m^−1^. The PANi nanorods provided a large pseudocapacitance and reduced volume changes upon cycling tests; the high conductivity could facilitate electron transport. Apart from rGO fibers, composite fibers can also act as the host fiber for active material loading [75,76]. Teng et al. reported a hierarchical fiber prepared by the in situ chemical polymerization of pyrrole on a microfluidic spun PEDOT:PSS/rGO fiber [75]. PPy was coated on the surface of the composite fiber through a strong π–π interaction. The ternary composite fiber retained a porous and wrinkled structure that could ensure fast ion transport, resulting in an ultrahigh areal specific capacitance of 983 mF cm^−2^.

#### 2.2.2. Films

In virtue of the planar structures of 2D nanosheets, it is feasible to obtain 2D macroscopic films of nanocomposites consisting of conducting polymers and 2D materials by various assembling strategies such as vacuum filtration, electrodeposition, and blade coating. 

**Figure 4:**
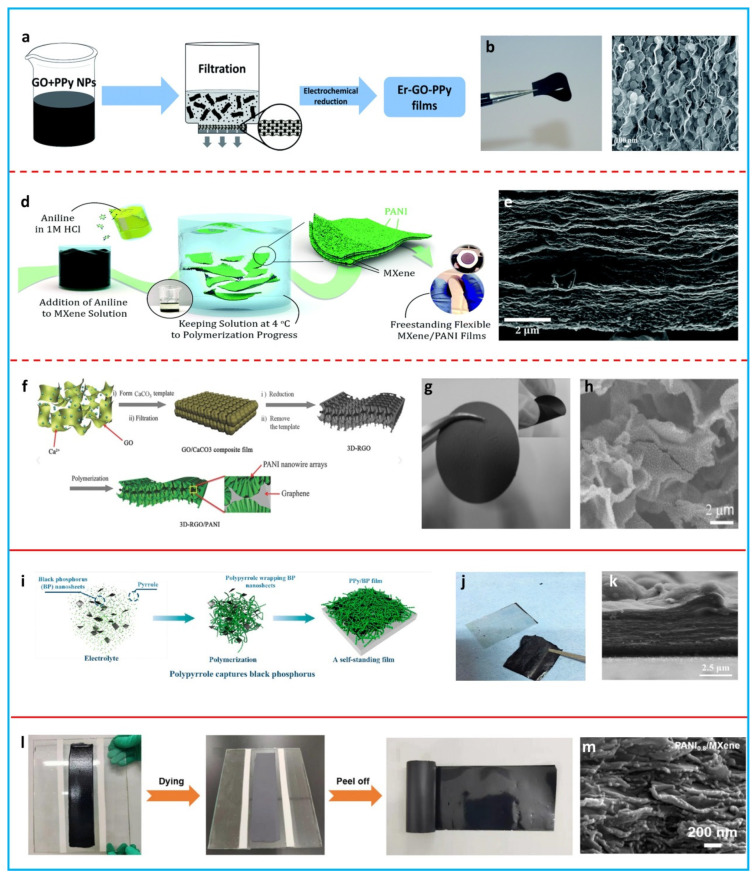
(**a**) Schematic procedure to produce PPy nanoparticles/rGO composite film. (**b**) Digital image of a flexible PPy nanoparticle/rGO composite film. (**c**) Cross-sectional SEM image of a PPy nanoparticle/rGO composite film. Reproduced from [77] with permission from the Royal Society of Chemistry. (**d**) Schematic of the process for the synthesis of a PANi/MXene composite film. (**e**) Cross-sectional SEM image of a PANi/MXene composite film. Reproduced from [84] with permission from the Royal Society of Chemistry. (**f**) Schematic process to fabricate a PANi nanowire array/rGO composite film. (**g**) Digital photo of a PANi nanowire array/rGO composite film. (**h**) SEM image of a PANi nanowire array/rGO composite film. Adapted with permission from [86]. Copyright 2013, Wiley-VCH. (**i**) Schematic fabrication flow of a PPy/BP composite film. (**j**) Digital image of a flexible PPy/BP composite film. (**k**) Cross-sectional SEM image of a PPy/BP composite film. Adapted with permission from [90]. Copyright 2018, American Chemical Society. (**l**) Scalable production of a PANi nanoparticle/MXene composite film. (**m**) Cross-sectional SEM image of a PANi nanoparticle/MXene composite film. Adapted with permission from [92]. Copyright 2021, Elsevier.

Vacuum filtration is a universal method to process solvent-dispersed materials into films. To obtain the macroscopic films of nanocomposites, two strategies have been proposed: ex situ synthesis and in situ synthesis. In ex situ synthesis, conducting polymers and 2D materials are separately prepared and hybridization is achieved by a simple blending and vacuum filtration [77,78,79,80,81,82]. This is a simple procedure to create composite films; however, it shows a disadvantage in the poor control of the interface between each component. Ge et al. prepared a free-standing PPy/rGO film by the vacuum filtration of a mixture of PPy nanoparticles and GO, followed by electrochemical reduction (Figure 4a) [77]. The PPy/rGO film was robust and highly flexible, with a high Young’s modulus of 11.77 MPa (Figure 4b). It can be clearly seen that the PPy nanoparticles were decorated between the layers of wavy and crumpled rGO sheets (Figure 4c). The composite film exhibited a large areal specific capacitance of 216 mF cm^−2^. They also prepared a PEDOT:PSS/MoS_2_ film using a similar method [78]. The obtained composite film showed an enhanced strength (23.5 MPa) compared with the neat MoS_2_ film (5.3 MPa), which could be attributed to the binding effect of PEDOT:PSS. The incorporation of PEDOT:PSS not only improved the mechanical properties, but also increased the conductivity. A large volumetric specific capacitance of 141.4 F cm^−3^ was obtained for this composite film. Zhang’s group demonstrated flexible aligned PEDOT:PSS/Mo_1.33_C MXene composite films prepared by the vacuum filtration of PEDOT:PSS and Mo_1.33_C MXene hybrid ink and a subsequent acid treatment [82]. The PEDOT:PSS confined between the MXene layers led to a fast reversible redox reaction and enhanced ion transport, leading to a high volumetric specific capacitance of 1310 F cm^−3^. In situ synthesis can provide a controlled interface between components at the molecular level to achieve a synergistic effect. Conducting polymers are generally formed in situ onto the nanosheets of 2D materials, followed by vacuum filtration [83,84] or are directly polymerized onto the vacuum-filtrated films of 2D materials to obtain the composite films [85,86,87,88]. Shu et al. prepared PPy nanofibers/graphene composite films by synthesizing PPy nanofibers onto liquid-phase exfoliated graphene sheets, followed by filtration [83]. The introduction of PPy nanofibers not only provided a large pseudocapacitance, but also enhanced the mechanical properties. The composite film delivered a specific capacitance of 161 F g^−1^. Beidaghi’s group reported a PANi/MXene composite film from the oxidant-free in situ polymerization of PANi on the surface of MXene nanosheets (Figure 4d) [84]. After vacuum filtration, a layer-structured composite film with excellent flexibility was obtained (Figure 4e). The PANi layer enhanced the ion-transport properties of the electrodes, leading to a high capacitance of 888 F cm^−3^, even in thick films. The second in situ synthesis approach involves the polymerization of conducting polymers on filtrated films of 2D material. Yu et al. produced a PPy/rGO composite film by the electropolymerization of PPy on a vacuum-filtrated rGO film [85]. The conformal coating of PPy on the rGO film increased the specific capacitance by four times to 237 F g^−1^ while maintaining the flexibility of the rGO film. However, the rGO sheets restacked during the filtration process in this work, resulting in limited ion transport. Creating porous networks can facilitate the access of ions to the internal surface. Meng et al. designed a porous flexible rGO film using CaCO_3_ as the template [86]. Then, PANi nanowire arrays were chemically polymerized onto the rGO sheets in situ (Figure 4f). The obtained composite film was flexible and could easily be bent (Figure 4g). The interconnected porous structure and the formation of PANi nanowire arrays could offer efficient pathways for ion diffusion (Figure 4h), thereby achieving a high-rate performance. The composite film showed a specific capacitance of 385 F g^−1^ and a capacitance retention of 89% when the current density increased from 0.5 to 10 A g^−1^. Similarly, Li et al. used polystyrene spheres as a template to construct flexible porous MXene films [87]. After the chemical polymerization of PANi, flexible porous PANi/MXene composite films were obtained. The composite film exhibited a high volumetric specific capacitance of 1632 F cm^−3^. An ultrahigh rate capability (827 F cm^−3^ at 5000 mV s^−1^) was achieved due to the interconnected porous structure.

Electrodeposition is a solution-processed approach to deposit composite films on conductive substrates [89,90]. Zhu et al. fabricated a flexible PPy/MXene composite film through a two-step electrodeposition method [89]. MXene was electrophoretically deposited onto fluorine-doped tin oxide glass, followed by the electrochemical polymerization of PPy. The formed PPy bonded with the MXene sheets through the hydrogen bonds between the N-H groups of pyrrole rings and the oxygen- or fluorine-containing terminal groups on the MXene sheets. The formed PPy/MXene composite film could be easily peeled off from the substrate. The MXene sheets could prevent dense PPy stacking and improve the stability of PPy backbones. The PPy/MXene composite film exhibited a volumetric specific capacitance of 406 F cm^−3^ and it showed a negative capacitance loss after 20,000 charging/discharging cycles. Luo et al. developed a laminated PPy/BP composite film using a one-step electrodeposition method (Figure 4i) [90]. The pyrrole monomer and BP nanosheets directly attached onto the conducting substrate and self-assembled into a film under a constant voltage. BP nanosheets were captured by PPy chains during the electrochemical process. PPy bonded with the BP nanosheets through the C-P bonds and hydrogen bonds between the N-H groups of pyrrole rings and hydroxyl attached to the BP nanosheets. The flexible composite film could be peeled off the substrate (Figure 4j) and it presented a laminated ordered structure (Figure 4k). The BP nanosheets not only guided a layer-by-layer assembly of PPy to facilitate ion transport, but also helped to enhance the structural stability of PPy. The film delivered a high volumetric specific capacitance of 551.7 F cm^−3^ and showed outstanding cycling stability over 10,000 charging/discharging cycles.

Although vacuum filtration and electrodeposition can prepare composite films in a facile way, the scalability is still limited by the dimensions of commercial vacuum filtration apparatus or conducting substrates. Thus, a scalable method for large-area composite films is highly desired to remedy the limitations of vacuum filtration and electrodeposition approaches. Blade coating was proposed to fabricate continuous composite films on a large scale. This approach utilized shear force to induce flake alignment. The proper rheological properties of the ink should be tuned for coating [91,92]. Wang et al. successfully fabricated a flexible PANi/MXene composite film with a layered structure using a mixture of PANi and MXene by blade coating (Figure 4l,m) [92]. The PANi nanoparticles not only delivered a high pseudocapacitance, but also reduced MXene stacking. The MXene sheets acted as dispersant, binder, and substrate for the PANi nanoparticles. The gravimetric capacitance of the composite film reached 560 F g^−1^.

### 2.3. Aerogels/Hydrogels

**Figure 5:**
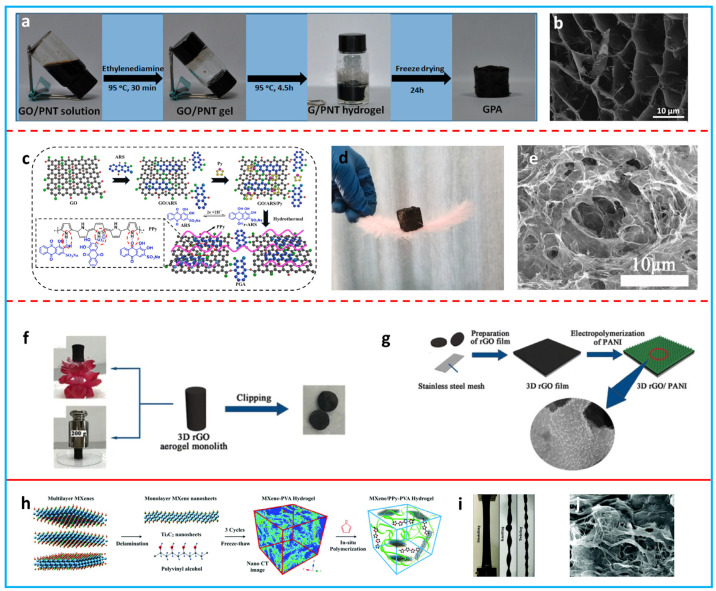
(**a**) Photos of the production of a PPy nanotube/rGO composite aerogel. (**b**) SEM image of the PPy nanotube/rGO composite aerogel. Adapted with permission from [93]. Copyright 2014, American Chemical Society. (**c**) Graphic illustration of the synthesis of a PPy/rGO composite aerogel. (**d**) Digital photo of the PPy/rGO composite aerogel standing on a feather. (**e**) SEM image of the PPy/rGO composite aerogel. Adapted with permission from [95]. Copyright 2022, Elsevier. (**f**) Schematic illustration for the fabrication process of 3D rGO aerogel slices. (**g**) Production of a PANi array/rGO composite aerogel by electropolymerization. Adapted with permission from [96]. Open access 2017, Springer. (**h**) Schematic of the synthesis of a PPy/MXene–PVA hydrogel. (**i**) Digital photos of the composite hydrogel at different mechanical deformations. (**j**) SEM image of the composite hydrogel. Reproduced from [99] with permission from the Royal Society of Chemistry.

Forming a porous 3D structure is an efficient way to prevent the restacking of 2D materials. The open pores can facilitate ion transport, which is highly desirable for high-rate FSCs [64,65]. The incorporation of conducting polymers into a 3D porous structure can provide a high capacitance. Three-dimensional composite aerogels/hydrogels with controlled porous structures have been prepared using various techniques [93,94,95,96,97,98,99]. GO is widely applied to prepare composite aerogels/hydrogels due to its good gelation ability under high-pressure water vapor. Ye et al. developed a 3D hierarchical PPy/rGO aerogel using GO and PPy nanotubes as the feedstock (Figure 5a) [93]. The GO acted as a surfactant to stabilize the PPy nanotubes. After a one-step hydrothermal reduction and freeze-drying, a highly porous PPy/rGO composite aerogel was obtained (Figure 5b). The composite aerogel showed a high specific capacitance of 253 F g^−1^. Different from adding conducting polymers into the GO dispersion, Han’s group added a pyrrole monomer into the GO dispersion to form a PPy/rGO hydrogel via a hydrothermal process (Figure 5c) [95]. The PPy/rGO composite aerogel was obtained after freeze-drying. It was light in weight and could stand on the surface of a feather (Figure 5d). A typical 3D porous network structure was observed for the composite aerogel (Figure 5e). This aerogel electrode provided an outstanding specific capacitance of 461 F g^−1^. Conducting polymers can also be polymerized onto rGO aerogels to produce composite aerogels. For instance, Yang et al. developed a PANi array/rGO composite aerogel via the electrochemical polymerization of a PANi array on a prepared rGO aerogel (Figure 5f,g) [96]. The obtained composite aerogel exhibited excellent electrochemical performance, with a specific capacitance of 432 F g^−1^. The gelation of MXene can be initiated by metal ions [100]; however, the obtained hydrogel is not strong enough for FSCs. The mechanical properties can be improved by integrating MXene with a hydrogel matrix such as polyvinyl alcohol (PVA). Zhang et al. prepared an MXene–PVA hydrogel using a cyclic freezing/thawing treatment [99]. PPy was chemically polymerized in a solution containing the MXene–PVA hydrogel to synthesize the composite hydrogel (Figure 5h). The obtained PPy/MXene–PVA hydrogel showed a remarkable strength of 10.3 MPa and could be stretched, knotted, and twisted (Figure 5i). The hierarchical structure of the composite hydrogel could promote electrolyte diffusion (Figure 5j), resulting in a high specific capacitance of 614 F g^−1^.

## 3. FSCs Based on Macroscopic Assemblies of Nanocomposites

Flexible electrodes play a key role in the electrochemical performance of FSCs; however, the importance of a rational design of the device configuration cannot be ignored. Recently, significant efforts have been devoted to develop varied architecture designs such as fiber-shaped, sandwiched, and interdigitated devices for FSCs.

### 3.1. Fiber-Shaped FSCs

**Figure 6:**
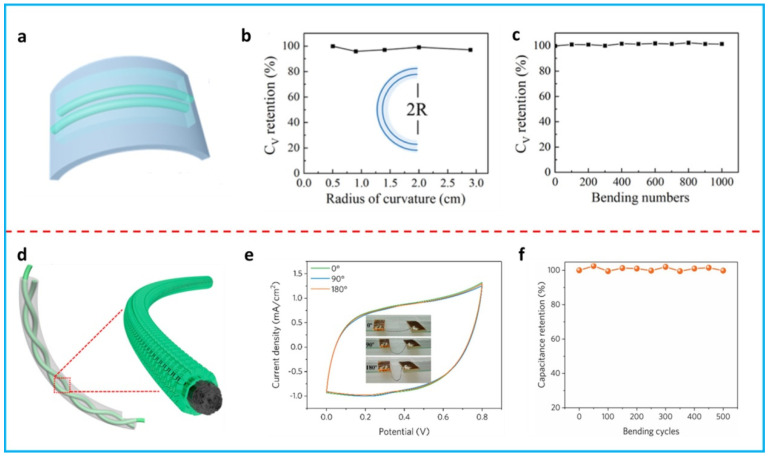
(**a**) Schematic of a fiber supercapacitor with parallel MoS_2_/PEDOT:PSS/rGO composite fiber electrodes. (**b**) Bending test of the fiber supercapacitor at different radii of curvature. (**c**) Repeated bending tests for 1000 cycles. Adapted with permission from [76]. Copyright 2023, American Chemical Society. (**d**) Schematic of a fiber supercapacitor with twisted PANi nanorod/rGO composite fiber electrodes. (**e**) CV curves of the fiber supercapacitor at different bending angles. (**f**) Cyclic bending tests for 500 cycles. Adapted with permission from [73]. Copyright 2019, American Chemical Society.

Fiber-shaped FSCs are generally built on fiber electrodes, which show incomparable advantages in being integrated with commercial textiles or garments [101]. Composite fiber electrodes based on conducting polymers and 2D materials are mainly assembled in parallel or twisted for fabricating fiber-shaped FSCs. In the parallel structure, two fiber electrodes are located in parallel and then the gel electrolyte is covered on the electrodes to obtain the fiber supercapacitors [66,70,76]. Razal’s group fabricated a fiber supercapacitor with PEDOT:PSS/MXene composite fibers in a parallel structure on a flexible polyethylene terephthalate (PET) substrate [66]. The potential window was limited to 0.6 V to avoid the oxidation of MXene. This fiber supercapacitor showed a volumetric specific capacitance of 361.4 F cm^−3^ at 2 mV s^−1^ (285.2 F cm^−3^ at 0.5 A cm^−3^). Zhou et al. fabricated a fiber FSC by assembling two MoS_2_/PEDOT/rGO composite fiber electrodes in parallel on PET, followed by covering the PVA–H_2_SO_4_ gel electrolyte (Figure 6a) [76]. When the fabricated supercapacitor device was bent to different radii of curvature or subjected to 1000 bending cycles, nearly no decay of the capacitance was observed (Figure 6b,c), suggesting outstanding flexibility and mechanical stability. 

A twisted structure can be obtained by twisting two fiber electrodes that are coated with a gel electrolyte. Compared with the parallel structure, a twisted structure can provide increased interfaces to enhance the electrochemical performance [102]. Zhang et al. reported a twisted-fiber supercapacitor with PANi nanorod/rGO composite fibers (Figure 6d) [73]. This fiber supercapacitor exhibited a high capacitance of 357.1 mF cm^−2^. Almost no changes in the cyclic voltammetry (CV) curves were observed when the fiber supercapacitor was bent to 180° (Figure 6e), indicating the high flexibility. Furthermore, it showed a capacitance retention of 99.8% after 500 bending cycles (Figure 6f), implying a high mechanical stability.

### 3.2. FSCs with a Sandwiched Structure

**Figure 7:**
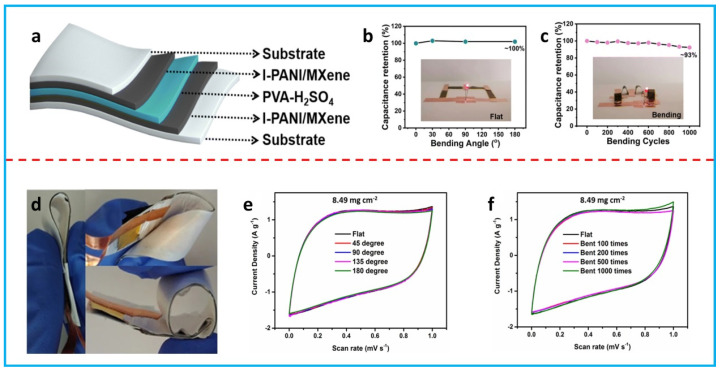
(**a**) Schematic of a flexible sandwiched supercapacitor with PANi/MXene composite film electrodes. (**b**) Bending tests at different angles. (**c**) Cyclic bending tests for 1000 cycles. Reproduced from [88] with permission from the Royal Society of Chemistry. (**d**) Digital photo of a flexible sandwiched supercapacitor with PEDOT:PSS/rGO composite film electrodes. (**e**) CV curves of the device at different bending angles. (**f**) CV curves of the device after different bending cycles. Adapted with permission from [91]. Open access 2015, Nature Publishing Group.

A sandwiched structure represents the most common device configuration for FSCs. A sandwiched flexible device is generally produced with a flexible electrode–electrolyte–flexible electrode configuration. Wang et al. assembled a flexible sandwiched supercapacitor device with PANi/MXene composite films and a PVA–H_2_SO_4_ gel electrolyte (Figure 7a) [88]. The specific capacitance derived from the device was 103 F g^−1^ at 1 A g^−1^. There was almost no capacitance loss when the device was bent from 0 to 180° (Figure 7b) and the capacitance retention was kept at ~93% after 1000 bending cycles (Figure 7c). Flexible current collectors should be added if the flexible electrodes are not conductive enough. Carbon cloth [81], stainless-steel mesh [78], and thin gold film [91] are widely employed as flexible current collectors due to their high conductivity and high flexibility. For example, Liu et al. fabricated a flexible device using PEDOT:PSS/rGO composite films attached to a gold film as flexible electrodes and PVA–H_3_PO_4_ as a gel electrolyte (Figure 7d) [91]. When the mass loading was 8.49 mg cm^−2^, the flexible device could deliver a high areal specific capacitance of 448 mF cm^−2^ at 10 mV s^−1^. The CV curves showed negligible changes when the device was bent at different angles and for 1000 bending cycles at 180° (Figure 7e,f). In addition to composite films, composite aerogels/hydrogels are also good candidates for FSCs. A flexible device with PPy/rGO composite aerogel electrodes and PVA–H_2_SO_4_ showed a high areal specific capacitance of 316 mF cm^−2^ at 1 mA cm^−2^ and it could provide a stable electrochemical performance at a bending angle of 135° [95].

### 3.3. Flexible Micro-Supercapacitors

**Figure 8:**
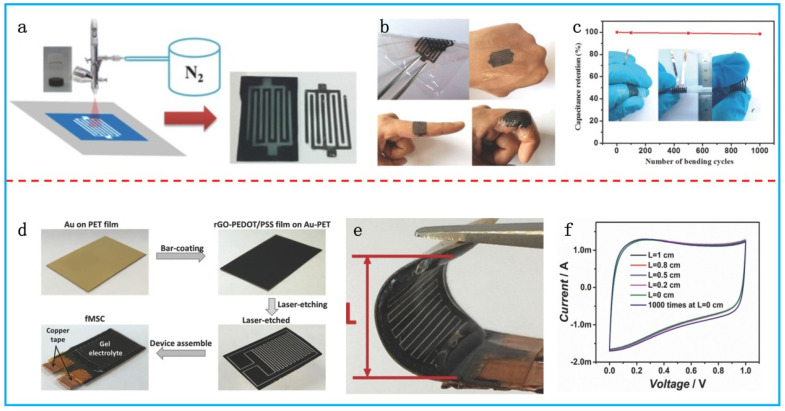
(**a**) Schematic illustration of the spray-coating method for the fabrication of an MSC with PEDOT:PSS/graphene electrodes. (**b**) Digital photos of the ultrathin MSC device. (**c**) Cyclic bending tests of the MSC. Adapted with permission from [104]. Copyright 2016, Wiley-VCH. (**d**) Schematic illustration of the fabrication process of an MSC with PEDOT:PSS/rGO films by laser etching. (**e**) Digital photo of the MSC device in a bending state. (**f**) CV curves of the MSC device at different bending states. Adapted with permission from [107]. Copyright 2016, Wiley-VCH.

Micro-supercapacitors (MSCs) with in-plane interdigitated electrodes have been proposed for the easy integration of other electronic components on the same substrate. The design of in-plane interdigitated electrodes offers several advantages: a high power density realized by the short distance between electrodes, a low risk of a short circuit, and easy electrolyte diffusion [103]. The fabrication strategies for micro-supercapacitors can be classified into two categories, depending on the deposition methods of the active materials, as either bottom-up or top-down. The bottom-up approach involves the formation of patterned electrodes using different methods such as spray-coating [104], electrodeposition [105], and mechanical pressing [106]. Liu et al. realized the direct printing of MSCs by spray-coating PEDOT:PSS/graphene hybrid ink through a shadow mask (Figure 8a) [104]. The fabricated MSC on a 2.5 μm PET substrate with PVA–H_2_SO_4_ delivered a maximum areal specific capacitance of 2 mF cm^−2^ at 5 mV s^−1^. The total thickness of the whole device was less than 5 μm and it could be attached to a human finger or other body parts (Figure 8b). The ultrathin device exhibited a stable performance with a capacitance retention of 98.5% after 1000 bending tests (Figure 8c). 

In the top-down approach, interdigitated micro-electrodes are obtained through the selective etching of the active materials. The etching methods include laser etching [107], plasma etching [108], mechanical scribing [109], and microfluidic etching [110]. For example, Liu et al. developed a flexible MSC by laser etching a PEDOT:PSS/rGO composite film (Figure 8d) [107]. A CO_2_ laser was used to create channels on the composite film to form micro-electrode arrays. The assembled flexible MSC with PVA–H_3_PO_4_ exhibited a remarkable areal specific capacitance of 84.7 mF cm^−2^ at 5 mV s^−1^. It was highly flexible with a slight change in CV curves when being repeatedly bent for 1000 cycles (Figure 8e,f).

## 4. Integrated Systems

**Figure 9:**
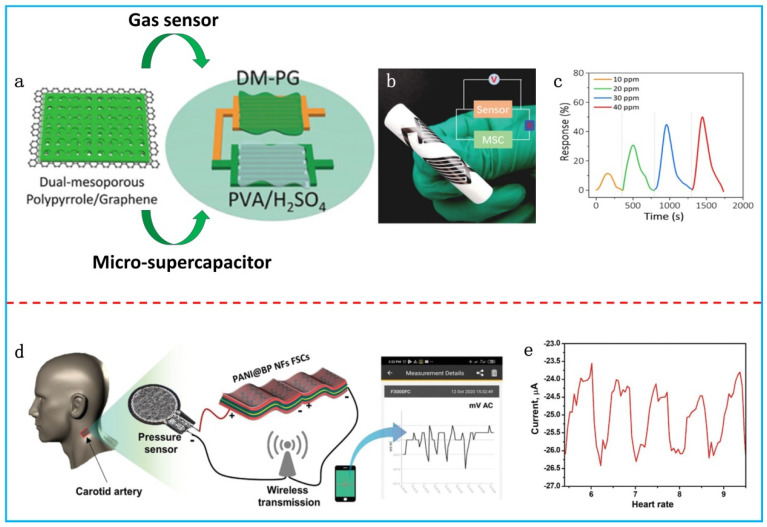
(**a**) Schematic diagram of an integrated gas-sensing system based on bi-functional dual-mesoporous PPy/rGO nanosheets. (**b**) Digital photo and equivalent circuit of the integrated system. (**c**) NH_3_ response curves of the integrated system. Adapted with permission from [111]. Copyright 2020, Wiley-VCH. (**d**) Schematic illustration of the pulse-monitoring system with a flexible supercapacitor based on a PANi/BP composite. (**e**) Heartbeat signal recorded using software. Adapted with permission from [112]. Copyright 2021, Wiley-VCH.

In recent years, there has been growing interest in developing a flexible self-powered sensing system by integrating various sensors with FSCs. A general strategy is to integrate each component on one substrate. Qin et al. fabricated an MSC–gas sensor-integrated system based on the bi-functional active material of hierarchical-ordered dual-mesoporous PPy/rGO nanosheets (Figure 9a) [111]. The dual-mesoporous PPy/rGO nanosheets showed an outstanding capacitance of 376 F g^−1^ and a superior sensing response to NH_3_ as low as 200 ppb. The flexible MSC with the composite nanosheets reached a maximum areal specific capacitance of 38 mF cm^−2^ at 1 mV s^−1^. The integrated system was highly flexible (Figure 9b). After charging for 100 s, the flexible MSC-powered gas sensor could quickly respond to NH_3_ at concentrations as low as 10–40 ppm (Figure 9c). 

In addition to the self-powered function, real-time data transmission is also essential for a wearable sensing system, especially for human health monitoring. Vaghasiya et al. fabricated an FSC with PANi/BP composites [112]. The flexible device was connected to a pressure sensor in a series and placed on the skin near to the carotid artery of the participant to record the periodic current signals (Figure 9d). The pulse signal generated by the FSC-powered press sensor could be monitored in real-time via a smartphone through the wireless data transmission of a Bluetooth multimeter (Figure 9e).

## 5. Conclusions and Prospects

We have presented the recent advances in the macroscopic assemblies of nanocomposites of conducing polymers and 2D materials for flexible supercapacitors. The relatively poor cycling stability of conducting polymers can be improved by the incorporation of 2D materials. Also, the conducting polymers can prevent the restacking of the nanosheets of 2D materials. Those nanocomposites exhibit superior performance compared with single components due to the synergistic effects. Macroscopic assemblies of the nanocomposites significantly determine the configurations of FSCs. The nanocomposites of conducing polymers and 2D materials can be developed into different macroscopic structures from 1D fibers to 3D aerogel/hydrogels, which can facilitate the fabrication of FSCs with varied configurations. The high mechanical strength of those macroscopic assemblies endows the resultant FSCs with excellent deformation tolerance, making them ideal power sources for integrated wearable electronics. 

For the future development of the nanocomposites of conducing polymers and 2D materials, in situ microscopic/spectroscopic techniques combined with multiphysics simulations are proposed for a deeper understanding of the electrochemical processes and structural evolutions of the nanocomposites within a supercapacitor system. Artificial intelligence (AI) can be employed to assist the design of the nanocomposites by optimizing the structures and synergistic effects through computational modelling and machine learning. It is also necessary to develop multifunctional nanocomposites to integrate more functions in one compact device. 

At the device level, novel fabrication techniques and manufacturing processes are in demand to promote large-scale production. The ultimate goal when fabricating FSCs is to realize an integrated wearable system. More attention should be paid to the integration methods of each component. Moreover, the efficiency of energy harvesting/conversion devices, the energy storage capability of FSCs, and the power consumption of the sensors should be well-balanced to achieve a practical self-powered system. With continuous efforts, we believe that the blooming of FSCs will undoubtedly accelerate the realization of a more intelligent world. 

## Data Availability

No new data were created or analyzed in this study. Data sharing is not applicable to this article.
